# A soft micron accuracy robot design and clinical validation for retinal surgery

**DOI:** 10.1038/s41378-025-01002-5

**Published:** 2025-09-09

**Authors:** Yiqi Chen, Xiangyu Guo, Xin Ye, Tong Jiang, Huan Chen, Jiafeng Yu, Ganglin Yang, Alois Knoll, Di Cui, Mingchuan Zhou, Lijun Shen

**Affiliations:** 1 Department of Ophthalmology, Key Laboratory of Precision Medicine for Eye Diseases of Zhejiang Province, Center for Rehabilitation Medicine,, Zhejiang Provincial People’s Hospital (Affiliated People’s Hospital, Hangzhou Medical College), Hangzhou, 314408 China; 2 Robotic Micron-nano Manipulation Lab, College of Biological Systems Engineering and Food Science, Zhejiang University, Hangzhou, 310058 China; 3 Hangzhou Dessight Biomedical Co. Ltd, Hangzhou, 310005 China; 4 Department of Informatics, Technical University of Munich, Munich, 85748 Germany

**Keywords:** Electrical and electronic engineering, Optical physics

## Abstract

Retinal surgery is one of the most delicate and complex operations, which is close to or even beyond the physiological limitation of the human hand. Robots have demonstrated the ability to filter hand tremors and motion scaling which has a promising output in microsurgery. Here, we present a novel soft micron accuracy robot (SMAR) for retinal surgery and achieve a more precise and safer operation. A remote center of motion (RCM) parallelogram structure with a double spring adaptive balancing mechanism is designed and optimized to achieve precise motion and safer operation. The deviation from the expected trajectory with manual operation and robot-assisted operation is 143.06 μm ± 91.27 μm vs 26.39 μm ± 13.22 μm, which has been significantly improved}. We evaluated the safety performance of SMAR in live animals. Furthermore, preliminary human clinical trials showed that the robot-assisted has less drift compared to the manual operation with 41.07 μm ± 20.78 μm vs 299.66 μm ± 85.84 μm. The visual acuity with LogMAR of cases showed higher improvement in the robot-assisted group preliminary, which for manual of 0.78 ± 0.44 vs robot-assisted 1.24 ± 0.70 with no statistically significant difference. This study provides promising options for robot-assisted with very experienced surgeons in the most challenging microsurgery. The system has the potential to effectively reduce the training curve of doctors and alleviate the shortage of ophthalmic surgeons, which is very important for rural areas and underdeveloped countries.

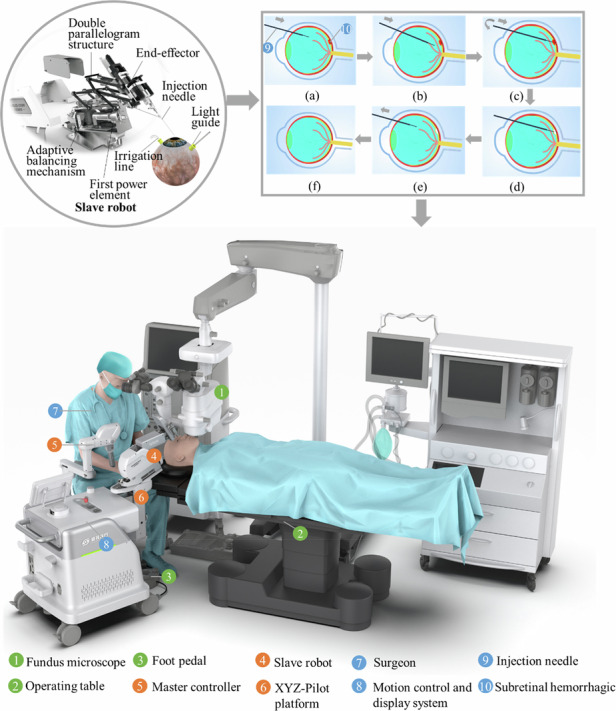

## Introduction

Retinal diseases are a leading cause of blindness worldwide, especially in developed countries. These diseases include diabetic retinopathy (DR), age-related macular degeneration (AMD), and retinal vein occlusion (RVO), affecting more than 100 million people vision health^1^. Retinal surgery is one of most important treatment to cure retinal disorders. However, this operation is treated as one of the most challenging microsurgery which is close to or even beyond the human limitation. During operation, three incision ports are created on the sclera in a circle by trocars around 3 mm away from the limbus. Afterward, the light source, surgical tool, and irrigation line are connected or inserted into the trocar port to illuminate the retina, operate the surgical procedure, and maintain the intraocular pressure^2–4^, respectively. The subretinal injection is a typical retinal surgery, which is clinically used to deliver nucleic acids, small molecules, macromolecules, viruses, and cells to the subretinal area for treating various retinal diseases such as subretinal hemorrhage, AMD, RVO, macular edema, and choroideremia^5–8^. The surgeon is required to inject a microcannula into a specific area of the translucent retina to a certain depth. The injection can create iatrogenic retinal detachment with an accuracy of no less than 10 μm^4,9,10^. During the operation, the surgeon not only needs to have a remote center of motion (RCM) to reduce the trauma to the sclera incision, but also needs to have a very steady hand to maintain the needle tip under the retina for a while to allow the liquid medicine to slowly inject to subretinal area. The delicate and precision operation skills could help to reduce the trauma and pressure damage to the fragile photoreceptors and retinal pigment epithelium (RPE)^11^. All those tissues and cells are delicate, fragile, and most probably irreversible for injuries. Only limited-talent surgeons can perform this procedure with a long training period and clinical experience, while the increasing number of patients and high-quality treatment expectations have proposed non-negligible challenges for the current ophthalmology system.

With the successful development and application of the Da Vinci Surgical System worldwide in the last 20 years, robot-assisted surgery has shown the capability to be a valuable and promising tool for achieving better clinical outcomes and patient satisfaction compared to traditional surgical techniques^12–14^. However, the Da Vinci Surgical System is targeted to laparoscopic surgery, therefore, its accuracy, layout design, and instruments are proven not suitable for eye surgery. To address the challenges of retinal surgery^10,15,16^, the researchers developed four typical surgical robots: (1) Hand-held surgical instrument^17–19^ has the advantage of intuitive operation. The surgeon holds the instrument directly but may lack the motion scaling and execution of the numerical motion. (2) Collaborative surgical robot is that the surgical instruments are controlled simultaneously by the surgeon’s hand and the robotic arm^20–22^. In this mode of operation, the surgical robot system can preserve the surgeon’s operation experience. However, it also introduces inertia and friction, which may limit its application in dynamic tasks. (3) Magnetic-controlled microrobot^23–25^ has a unique advantage in targeted drug therapy since it has a better degree of freedom intraocular. However, the microsurgery it can perform is limited due to its small interaction force generated by a magnetic field. (4) Teleoperation systems^26–28^ are the most popular operation mode in robot-assisted surgery with the benefit of providing tremor filtering and motion scaling^29^. These systems are known to be the most successful in commercial products. However, robots designed for general surgery apart from their significant footprints, do not have sufficient precision for retinal surgery^30^. With this operation mode, the robotic retinal dissection device with teleoperation functionality performed the world’s first robotic retinal surgery^31^ proving the feasibility and safety concept of the robot-assisted eye surgery. There are several trade-offs between mechanical design for stroke, actuating force, accuracy, and the power-to-weight ratio among these options, while keeping micron accuracy. The safety of the robotic system can also be improved if the system can behave softly.

In this research, we present a soft micro accuracy robot (SMAR) for retinal surgery and aim for more stable and safer operation. An RCM parallelogram structure with a double spring adaptive balancing mechanism is designed and optimized to avoid motion interference, achieving more precise motion and safer operation. The position precision of the SMAR is 5.56 μm, and trajectory deviation is reduced by 81.85% compared with the human hand. Animal and human clinical trials demonstrated that the proposed methods minimize the influence of hand tremors and motion impact on the instrument. Eye surgical robots can help surgeons break through the limits of physiological operation, allowing surgeons to focus more on intraoperative decision-making instead of skills on delicate operation and tremor suppression. This study provides promising options for alleviating the shortage of experienced surgeons and addressing the pressing problem of disparities in medical resources.

## Results

### Design of the SMAR

**Fig. 1:**
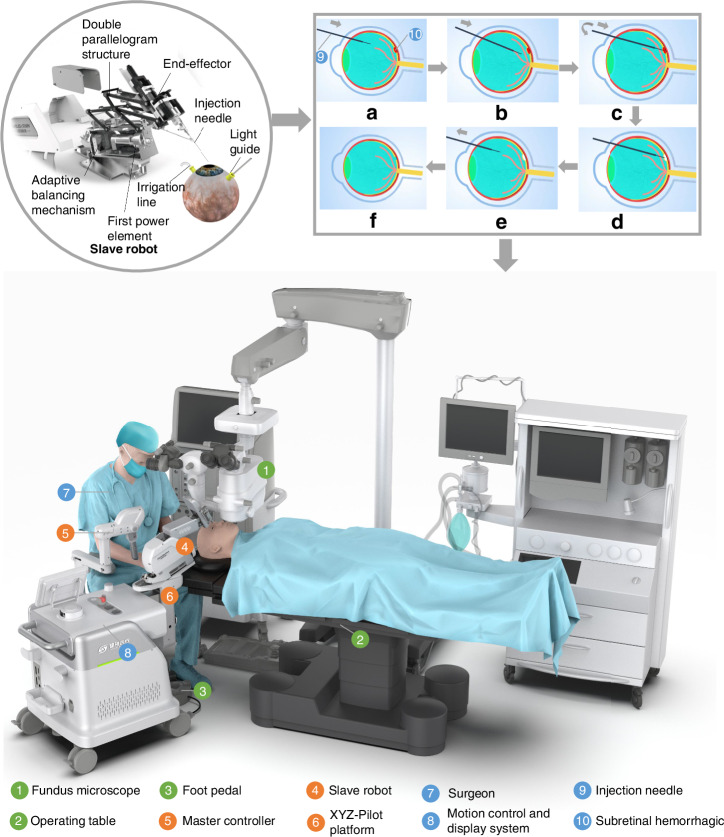
SMAR system overview for retinal surgery. The SMAR consists of a slave robot, a robot master controller, and a motion control and display system. During the operation, the doctor observes the enface image of the retina in real-time through the microscope, then controls the master operator to drive the slave robot arm to complete the retinal operation. As shown in procedure (**a**) the needle is inserted through the trocar into the vitreous body and approaches the lesion (**b**). Then the robot is controlled to puncture into subretinal space (**c**) and the drug can be delivered slowly to create subretinal bubbles (**d**). Afterward, the needle is retrieved from intraocular (**e**, **f**) (see Supplementary Video 1)

**Fig. 2:**
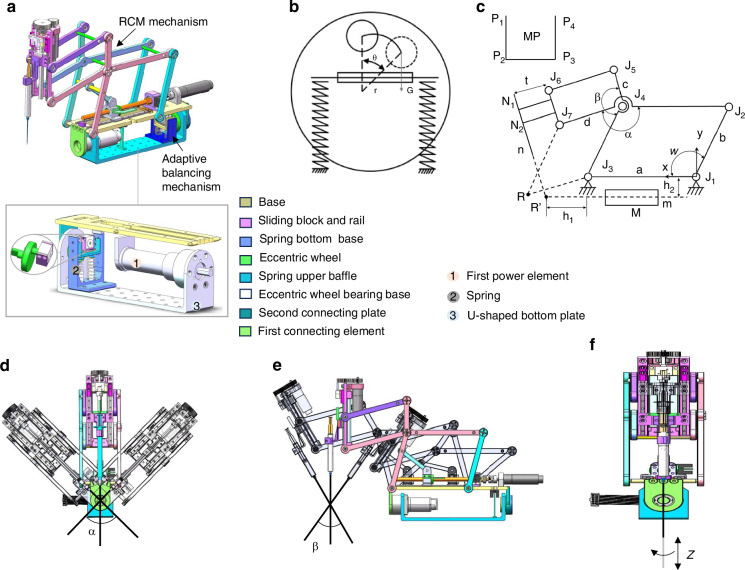
Design and kinematics of slave robot. **a** 3D model of the slave robot. **b** Kinematic diagram of the adaptive balancing mechanism. Deflection angle: 0°–45°. **c** The kinematics analysis of the slave robot. **d**–**f** The motion axes for the *α*-axis, *β*-axis, and *Z*

The SMAR is designed to treat retinal fundus diseases with more accuracy and safety (Fig. 1). The SMAR consists of a slave robot, an operating table, a foot pedal, a motion control and display system, a robot master controller, and a seat. During the operation, the surgeon observes the enface view of the retina in real-time through the microscope, then controls the master controller to remotely drive the slave robot arm to complete the corresponding surgical actions. The motion control system is responsible for processing the motion information of the master controller and sending out the control signal to complete the motion control of the slave robot. The slave robot mainly consists of three parts: RCM mechanism, adaptive balancing mechanism, and RCM repositioning mechanism (Fig. 2a). An RCM parallelogram structure with a double spring adaptive balancing mechanism is designed and optimized to avoid motion interference, achieving more precise motion, and safer operation. The RCM point is the R point in Fig. 2c. The designed RCM mechanism has four degrees of freedom (DOF), which are the in-plane yaw *α*, out-of-plane pitch β, and translation and rotation along the instrument axis *Z* (Fig. 2d–f) (see Supplementary Video 2).

The design of the RCM mechanism mainly has the following three requirements. Firstly, the RCM point of the mechanism overlaps with the target RCM point. Secondly, the R’ position falls into the intersection of the M-axis of the first power element and the axis *N*_1_*N*_2_ of the instrument. According to the geometry, the *R’* coordinate is (*h*_1_ + $a$, -*h*_2_), where *h*_1_ is the *X*-axial projection distance from *R*’ to the node *J*_3_. The position coordinates of *R* (*x*, *y*) can be expressed as:

1 $$\left\{\begin{array}{l}x=a-{dcos}\alpha \\ y={dcos}\alpha \end{array}\right.$$

The relationship between *J*_5_(*x*_1_, *y*_1_), *J*_6_ (*x*_2_, *y*_2_), and *N*_1_ (*x*_3_, *y*_3_) is as follows:

2 $$\left\{\begin{array}{c}{{\boldsymbol{J}}}_{{\bf{1}}}{{\boldsymbol{J}}}_{{\bf{5}}}={{\boldsymbol{J}}}_{{\bf{1}}}{{\boldsymbol{J}}}_{{\bf{2}}}+{{\boldsymbol{J}}}_{{\bf{2}}}{{\boldsymbol{J}}}_{{\bf{4}}}+{{\boldsymbol{J}}}_{{\bf{4}}}{{\boldsymbol{J}}}_{{\bf{5}}}\\ {{\boldsymbol{J}}}_{{\bf{1}}}{{\boldsymbol{J}}}_{{\bf{6}}}={{\boldsymbol{J}}}_{{\bf{1}}}{{\boldsymbol{J}}}_{{\bf{2}}}+{{\boldsymbol{J}}}_{{\bf{2}}}{{\boldsymbol{J}}}_{{\bf{4}}}+{{\boldsymbol{J}}}_{{\bf{4}}}{{\boldsymbol{J}}}_{{\bf{5}}}+{{\boldsymbol{J}}}_{{\bf{5}}}{{\boldsymbol{J}}}_{{\bf{6}}}\\ {{\boldsymbol{J}}}_{{\bf{1}}}{{\boldsymbol{N}}}_{{\bf{1}}}={{\boldsymbol{J}}}_{{\bf{1}}}{{\boldsymbol{J}}}_{{\bf{2}}}+{{\boldsymbol{J}}}_{{\bf{2}}}{{\boldsymbol{J}}}_{{\bf{4}}}+{{\boldsymbol{J}}}_{{\bf{4}}}{{\boldsymbol{J}}}_{{\bf{5}}}+{{\boldsymbol{J}}}_{{\bf{5}}}{{\boldsymbol{J}}}_{{\bf{6}}}+{{\boldsymbol{J}}}_{{\bf{6}}}{{\boldsymbol{N}}}_{{\bf{1}}}\end{array}\right.$$

3 $$\left\{\begin{array}{c}{x}_{1}=a+{bcos}\omega -{ccos}(\beta +\omega )\\ {y}_{1}={bsin}\omega -{csin}(\beta +\omega )\\ {x}_{2}=a+{bcos}\omega -{dcos}\alpha -{ccos}(\beta +\omega )\\ {y}_{2}={bsin}\omega -{dsin}\alpha -{csin}(\beta +\omega )\\ {x}_{3}=a+{bcos}\omega -\left(d+t\right)\cos \alpha -{ccos}(\beta +\omega )\\ {y}_{3}={bsin}\omega -\left(d+t\right)\sin \alpha -{csin}(\beta +\omega )\end{array}\right.$$

Finally, as the change of ω, the trajectories of *J*_5_, *J*_6_, and *N*_1_ on the mechanism do not interfere with the microscope region (*P*_1_, *P*_2_, *P*_3_, *P*_4_). During the movement of the mechanism, the end points can cover the focal area on the surface of the fundus. Assuming that the focus region is *A*, the forward kinematics solution accessibility region of the robot is *F* (*J*) ∋ *A*. The constraints of the linkage (*a*, *b*, *c*, *d*, *α*, *β*, *t*) are obtained with constraints as follows:

4 $$\left\{\begin{array}{c}R={R}^{{\rm{\mbox{'}}}}\\ F(J)\ni A\\ {J}_{5},{J}_{6},{N}_{1}\,\notin\, {MP}\end{array}\right.$$

To better balance dynamic load and improve the accuracy and safety of the SMAR, a double spring adaptive balancing mechanism is specifically designed, which comprises an eccentric wheel, an eccentric bearing base, a sliding block and rail, double parallel springs, and a spring base. These components are sequentially connected and integrated directly below the second power element. The second connecting plate is connected to the base, which moves with the base to drive the eccentric wheel movement. The moment inertia of the base is converted by the eccentric wheel to the right and left or up and down movement of the eccentric bearing seat and the side guide rail. Thus, the base can move smoothly. At the same time, the design can reduce the load of the first power element and buffer the inertia force generated in the process of acceleration and deceleration rotation, which can achieve more accurate control of the instrument. In the case of motor power failure, the damping of the motor and the buffer of the balance mechanism can make the robot remain stationary at any angle. It will not be affected by the gravitational inertia of the robot, which can reduce the load variation for the motor. Therefore, it makes the power output of the first power component smoother and more accurate. Thus, it brings more precision and smoothness to the operation of the surgical instrument. The joint flexible torque can be calculated by the current loop as the output torque on the motor. The soft behavior of the robot is realized through giving the limitation of the torque controller to improve the safety and stability of the robot during the operation. The output torque *T* is expressed as,

5 $${T=K}_{\Phi }{\Phi }_{i}$$

where *T* < *T*_0_, *T*_0_ is set to the maximum output torque of the motor. *K*_Φ_ is the torque coefficient of the motor and ${\Phi }_{i}$ is the current of the motor. When load *G* is loaded on the motor (Fig. 2b), the motor output torque M is,

6 $$\begin{array}{l}M={M}_{{load}}+{M}_{{mom}}-{M}_{k}={rG}\sin \theta\\\qquad+\left({J}_{{motor}}+{J}_{{load}}\right){\omega }^{{\rm{\mbox{'}}}}-2{kr}(1-\cos \theta )r\end{array}$$

where $M$ load is the torque of the *G*, ${M}_{{mom}}$ is inertia moment, ${M}_{k}$ is the torque of the springs, *θ* is the deflection angle, ${\omega }^{{\rm{\mbox{'}}}}$ is the angular acceleration, *k* is the elastic coefficient, ${J}_{{motor}}$ and ${J}_{{load}}$ are the rotational inertia of the motor and load, and r is the radius of the eccentric wheel. *M* is equal to *T*. The RCM repositioning mechanism is a linear XYZ-Pilot platform to relocate the RCM point. The slave robot is mounted on the RCM repositioning mechanism so that the surgeon can adjust the XYZ-Pilot platform to align the needle tip to the entry point of the trocar on the sclera.

### Characterization of the SMAR

**Fig. 3:**
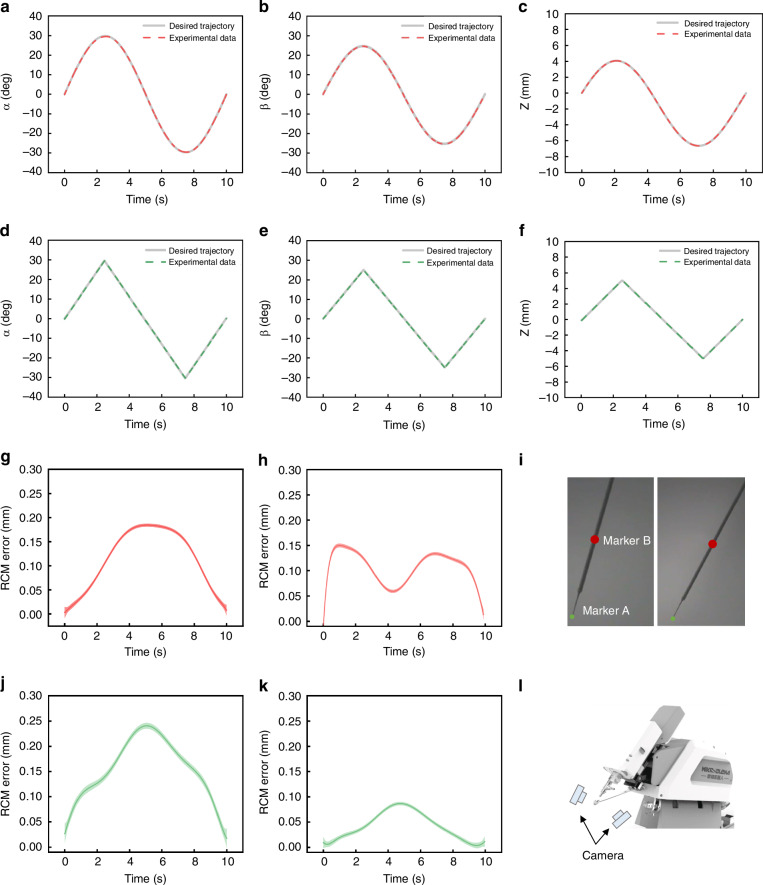
Characterization of the SMAR. The motion precision of the SMAR was evaluated by sinusoidal trajectory and linear trajectory tests, respectively. **a**–**c** Experimental sinusoidal trajectory and experimental data. The amplitudes of *α* and *β* were 30°, 25°, and 5 mm, respectively. **d**–**f** Experimental linear trajectory and experimental data. The amplitude of *α* and *β* were 30°, 25°, and 5 mm, respectively. **g**, **h** RCM error during sinusoidal motion. **i** Microneedle mark points. **j**, **k** RCM error during linear motion. **l** Two cameras were placed vertically on the front and side of the SMAR to capture the trajectories of two markers (**A** and **B**)

Precision measurement. The motion precision of the SMAR was evaluated by sinusoidal trajectory and linear trajectory tests, respectively. The encoder experimental data were compared with the desired trajectory data. The results showed that the root-mean-square (RMS) precision of the sinusoidal motion *α*, *β*, and *Z* were 0.14° ± 0.08°, 0.13° ± 0.06°, and 5.43 ± 0.66 μm, respectively (Fig. 3a–c). The RMS precision of the linear motion angles *α*, *β*, and *Z* were 0.16° ± 0.08°, 0.13° ± 0.002°, and 5.56 ± 0.14 μm, respectively (Fig. 3d–f).

RCM error assessment. The RCM error was evaluated by the marker B movement trajectory, which was defined as the deviation between the marker position and the desired RCM position. Two cameras were placed vertically on the front and side of the SMAR to capture the trajectories of two markers (A and B) (Fig. 3i, l). The visual-tracking result showed that the RCM errors of the sinusoidal trajectory *α* and *β* were 0.11 mm and 0.11 mm, respectively (Fig. 3g, h). The RCM errors of the linear trajectory *α* and *β* were 0.15 mm and 0.04 mm, respectively (Fig. 3j, k) (see Supplementary Video 3). Literature^26,32,33^ reported that the maximum motion error was limited to what was within clinical tolerance referred to as 3 mm free-hand error performance. The smaller RCM error can effectively reduce the trauma on the sclera. So, the motion error ensures that the designed SMAR can achieve safer and less trauma operation.

Motion range. The motion range of the SMAR was tracked by time-lapse photography. The results showed that the motion ranges of *α*, *β*, and *Z* were 90°, 60°, and 40 mm, respectively. Literature^34^ reported the motion ranges from the clinical requirement of *α*, *β*, and *Z* were 50°, 56°, and 30 mm, respectively. Obviously, the SMAR can meet the requirements.

**Fig. 4:**
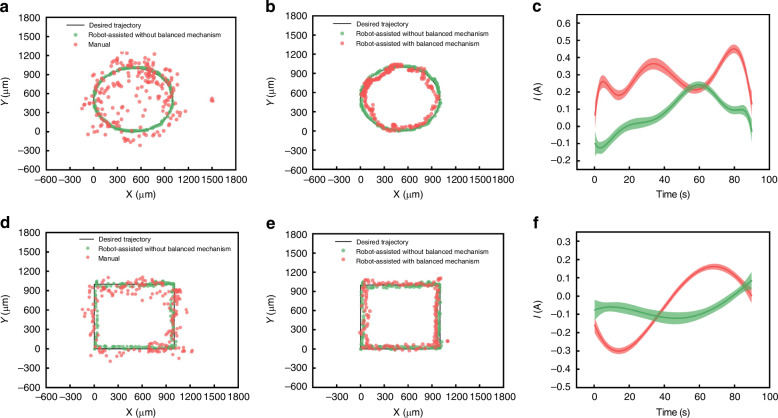
Teleoperation test. **a**, **b** Circle trajectory tracking. The experimental data of manual operation, robot-assisted operation with balanced mechanism, and robot-assisted operation without balanced mechanism compared with the desired trajectory. **c** The motor current of circle trajectory. The green line represents the motor current of the robot-assisted operation with balanced mechanism, and the red represents the motor current of the robot-assisted operation without balanced mechanism. **d**, **e** Square trajectory tracking. The experimental data of manual operation, robot-assisted operation with balanced mechanism, and robot-assisted operation without balanced mechanism compared with the desired trajectory. **f** The motor current of square trajectory

Teleoperation test. Two different sample tasks were designed to demonstrate the device’s ability to perform fine operations under teleoperation, square and circle tests respectively (Fig. 4). The operator performed master controller to control the SMAR to track the circle with a diameter of 1 mm or the square with a 1 × 1 mm, and the motion trajectory of the micromanipulator was recorded by the microscope. The operator repeats the above tasks by hand (see Supplementary Video 4). As shown in Fig. 4a–f, the circle trajectory error of the proposed method was 13.54 μm ± 8.21 μm, which was significantly better than the manual operation (143.06 μm ± 91.27 μm). The square trajectory error of the proposed method was 26.39 μm ± 13.22 μm, which was significantly better than the manual operation effect (98.32 μm ± 58.27 μm). The results showed that the proposed SMAR was superior to manual operation on both circle and square tests. In addition, we also designed a comparison test with balanced and unbalanced devices. The result showed that the operating error with balanced device (square test and circle test) was lower than that without balanced device (56.64 μm ± 31.27 μm vs 61.43 μm ± 33.57 μm, respectively). The proposed mechanism can not only effectively decrease the tracking error, but also significantly reduce the motor current which can improve the operation accuracy and safety (Fig. 4c, f).

### Evaluation of live animal experiment

Ten pigs were used to compare the safety and efficacy of subretinal injection by the manual and robot-assisted groups. Fast the animals overnight before administration, and the first administration was marked as D1. Compound sodium chloride was a single dose (100 μL/eye) subretinal injected manually on the left eye and assisted with the ophthalmic surgical robot on the right eye. Duration distribution for subretinal operation in each step, including time for operation, puncture, and injection in manual and robot-assisted group was recorded and evaluated by reviewing the footage. Operation time was defined as the period from the moment the microcannula entered the trocar until the needle was withdrawn from the trocar after completing the intraocular operation. Each retinal puncture time was defined as the period from the moment of microcannula touched the retinal target puncture site until it reached the target depth. Each drug injection time was defined as the period from the moment the microcannula punctured into the cavity and injected the drug under the retinal nerve epithelium layer. Groups and solvent dose settings were shown in the supplementary information.

**Fig. 5:**
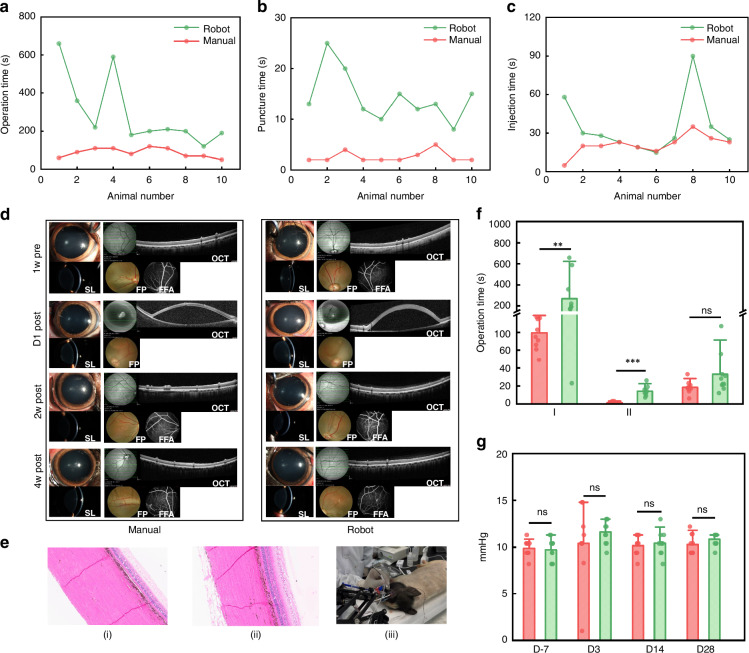
Experiment on live animals. **a** The mean time of each operation. **b** The mean time of each puncture. **c** The mean time of each injection. **d** Results of postoperative eye examination. OCT optical coherence tomography, SL slit lamp, FP fundus photography 1-week pre-operation and 1 day, 2 weeks, 4 weeks post-operation, FFA fundus fluorescein angiography, ERG electroretinography examination 1-week pre-operation and 2 weeks, 4 weeks post-operation. **e** (i)-(ii) Histological evaluation results on day 28 post-operation. (iii) Animal operation. **f** Total puncture and injection time analysis of all experimental groups. **g** Intraocular pressure. D-7: Intraocular pressure 1-week pre-operation. D3: Intraocular pressure 1-week post-operation

The injection success rate with robot-assisted was 100%, and 80% manually, for two out of the ten injections leaking in the vitreous cavity. The animal learning curve was analyzed in the animal experiments within ten trials in the robot-assisted group compared with the manual group, which was shown in Fig. 5. The time trend distribution showed that the mean time of each operation (Fig. 5a), and the mean time of each puncture (Fig. 5b) in the robot-assisted group was gradually shortened, and gradually converge with the manual group (Fig. 5c). Compared with the manual group, the mean time of each injection in the robot-assisted group was equivalent in five cases, and the convergence of the distribution remains high. Thus, the analysis revealed that although the mean time of operation increased in the robot-assisted group, the time of each retinal puncture had a decreasing trend and achieved almost equal time after ten trials.

Clinical analysis was also carried out before and after the operation for safety evaluation. During the trial period, no significant abnormalities were observed in the clinical observation, body weight, and food intake of the animals. No significant abnormalities were observed in the animals’ eyes before the operation. After the operation, conjunctival hyperemia and conjunctival edema were observed, with no significant differences in the incidence and severity between the manual and robot-assisted groups (Fig. 5d). During the trial period, there were no significant abnormalities in intraocular pressure in the animals’ eyes compared to before administration. Histological evaluation results on day 28 post-operation showed no differences between manual and robot-assisted groups (Fig. 5e). The distribution of duration for subretinal operation in manual and robot-assisted groups was shown in Fig. 5f. Compared with the manual group, the mean time for retinal operation (79.40 s vs 320.80 s) in the robot-assisted group was significantly increased (*P* < 0.01), the mean time for each puncture (1.50 vs 14.10 s) was significantly increased (*P* < 0.001), the mean time of each injection (18.40 s vs 32.90 s) was non-significantly increased (*P* > 0.05) in the robot-assisted group. Individual intraocular pressure data was shown in Fig. 5g.

### Clinical assessment of the SMAR

**Fig. 6:**
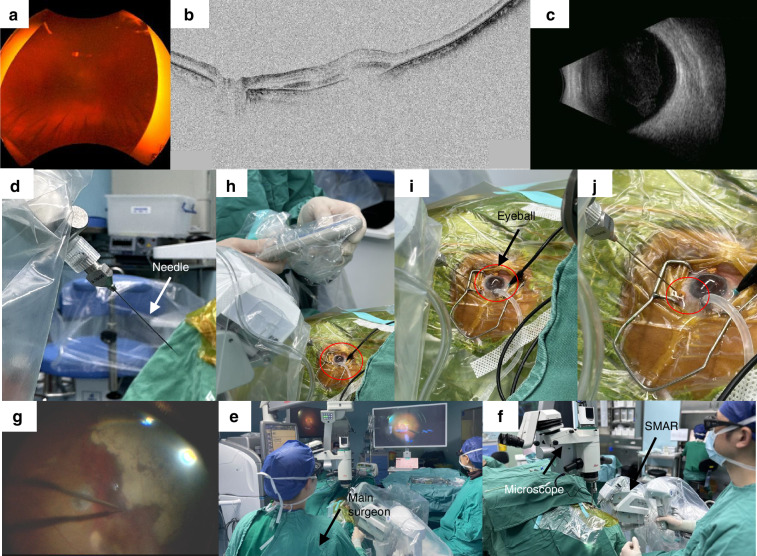
Clinical assessment. **a**–**c** Left eye bottom examination image of a patient with decreased left eye vision for 1 month. Ultra-widefield color fundus image showing vitreous hematocele. Coherent light tomography image shows a subretinal hyperreflective focus (arrow) in the macula, with a local retinal pigment epithelium bulge. Ocular B-ultrasound images showed vitreous hematoma and abnormal bulb echo. **d** The end-effector detail diagram. A 41G hyperfine microcannula is connected to the 1 ml syringe containing the drug with a soft tube. **e**–**j** Doctors operate microsurgical robots for subretinal manipulation

Ten patients with subretinal hemorrhage (submacular hemorrhage) were involved from January 11, 2023 to December 20, 2023. All the patients signed the informed consent and underwent the preoperative examinations. Vitrectomy combined with robot-assisted retinal puncture was performed under general anesthesia. As shown in Fig. 6, the surgical process was as follows: (1) After vitrectomy, the subretinal hemorrhage in the macular area involving the fovea could be seen (Fig. 6a). With the help of a real- time observation using the OCT (VG200, Vision Microimaging Technology Co., Ltd, Henan, China), the lesion was considered to be polypoidal choroidal vasculopathy (PCV) (Fig. 6b, c). (2) The slave robot was placed on the temporal side, and a 41G hyperfine microcannula was connected to a 1 ml syringe, which contained the recombinant tissue plasminogen activator (rt-PA) solution. The syringe was installed at the end of the robot arm of the slave robot (Fig. 6d). (3) The SMAR system assisted in the subretinal injection (Fig. 6e–j): The robot master controller was controlled by the operator to control the position of the needle, which moved near the trocar. Through the robot master controller, the microcannula was controlled and inserted through the trocar into the eye. The retinal puncture point was planned and located under the operating microscope. The microcannula position can be adjusted in real-time. Then, the needle reached the retinal puncture location and finished the puncture process, and stopped between the retinal neurosensory layer and the RPE layer. (4) Subretinal drug injection: 0.2 mg/ml of rt-PA solution was injected into the space between the retinal nerve layer and the RPE layer at a uniform rate. The effective drug injection could be observed by the intraoperative OCT (Leica Proveo 8 Enfocus, Leica Camera AG, Wetzlar, Germany). (5) When the injection was finished, the doctor operated the master controller to control the needle to withdraw from the retina and retrieve it from the eye. Finally, a gas-liquid exchange of the vitreous cavity was completed manually. Details of postoperative patient recovery were listed in Table 1.

**Table 1:** Clinical details of patients

Patient	Method	Eye	Age	Disease type	Puncture time (min)	Injection time (min)	Injection volume (ml)	Blood displaced	PRVA (LogMAR)	POVA (LogMAR)
R1	Robot	Left	65	PCV	11.00	5.26	0.20	Yes	2.30	1.00
R2	Robot	Right	60	PCV	5.90	2.51	0.25	Yes	2.60	0.82
R3	Robot	Left	76	PCV	5.23	3.51	0.10	Yes	2.30	0.70
R4	Robot	Right	53	PCV	3.86	6.91	0.15	Yes	0.51	0.30
R5	Robot	Left	63	RAM	3.60	2.45	0.05	Yes	0.82	0.40
M1	Manual	Left	67	PCV	0.33	0.95	0.15	Yes	2.30	1.00
M2	Manual	Left	71	PCV	0.13	0.50	0.10	Yes	1.00	0.22
M3	Manual	Right	71	PCV	0.28	4.28	0.15	Yes	2.00	1.85
M4	Manual	Left	66	PCV	0.23	2.93	0.15	Yes	0.60	0.22
M5	Manual	Right	44	PCV	0.48	2.7	0.15	Yes	1.30	0.52

LogMAR (Logarithm of the Minimum Angle of Resolution) is a standardized unit used to measure visual acuity, commonly applied in the field of ophthalmology. It provides a way to quantify vision by logarithmically converting the results of visual tests, particularly used to describe visual sensitivity during eye examinations. Smaller LogMAR values indicate better vision, while larger LogMAR values correspond to poorer vision. PRVA: Pre-operative visual acuity, POVA: Post-operative visual acuity. Post-operative visual acuity was defined as corrected visual acuity at two months after surgery

**Fig. 7:**
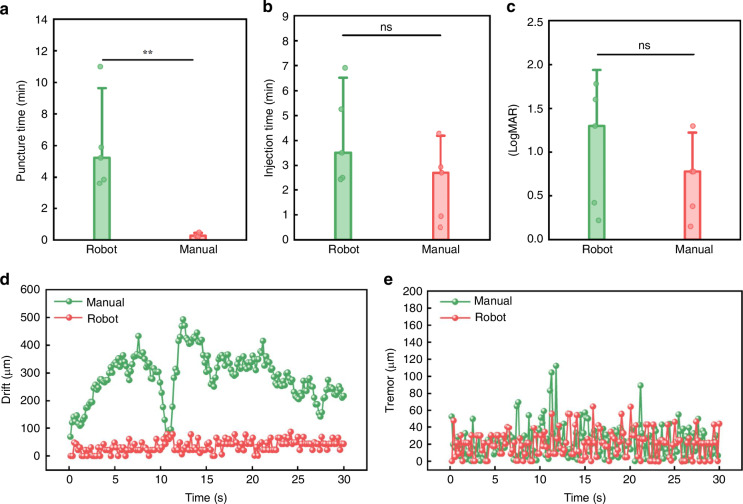
Comparison of clinical outcomes between robot-assisted and manual approaches. **a** Puncture time. **b** Injection time. To puncture time (0.31 ± 0.13 min vs 6.50 ± 3.00 min, *P* = 0.003) and subretinal drug injection time (2.66 ± 1.54 min vs 4.48 ± 1.93 min, *P* > 0.05), human hand efficiency was significantly faster than robot-assisted puncture. **c** Preoperative and postoperative visual acuity changes. There was no statistically significant difference between the robot-assisted method and the manual method. **d**, **e** Error. Manual drift and tremor vs robot-assisted drift and tremor

**Fig. 8:**
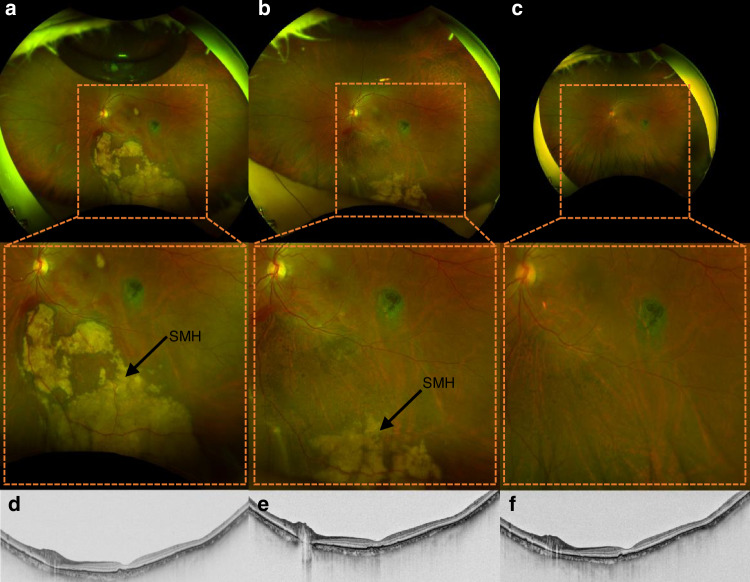
Post-operative assessment. **a**–**f** Ultra-widefield color fundus images showed that submacular hemorrhage (SMH) was further significantly reduced than 1 week, 1 month, and 4 months after surgery, and OCT showed that SMH lesions were significantly smaller than 4 months after surgery, and the intraocular fluid disappeared. Four months after surgery, the naked visual acuity in the left eye was 0.03, the corrected visual acuity was 0.1, and no other complications were observed

**Fig. 9:**
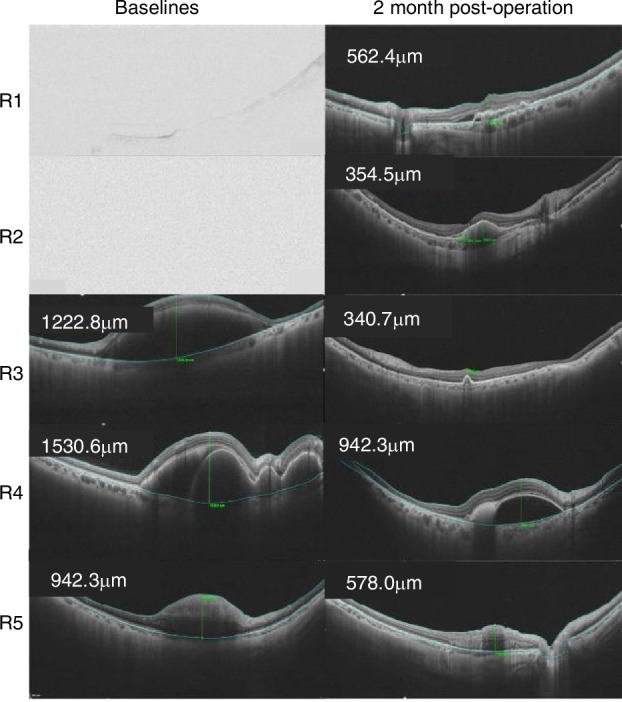
Pre-operative and post-operative OCT. All five patients had subretinal hemorrhage in the central fovea, and R1 and R2 were accompanied by vitreous hemorrhage. The hemorrhage in the vitreous cavity was cleared after vitrectomy in R1-R2 patients. Subretinal hemorrhage in the central fovea subsided in all patients after surgery

As shown in Table 1, the research included 9 cases attributed to PCV and 1 case to retinal arterial microaneurysm (RAM). Among these cases, five cases involved robot-assisted subretinal injection while 5 cases involved manual subretinal injection. The average age of the patients was 64.22 ± 9.38, with 5 males and 5 females. At the final follow-up, all cases showed varying degrees of improvement in LogMAR scores. The robot-assisted group had a mean LogMAR of 1.24 ± 0.70, while the manual group had a mean LogMAR of 0.78 ± 0.44. Although the difference between the two groups was not statistically significant (*P* > 0.05), the robot-assisted group demonstrated superior outcomes compared to the manual group, suggesting a trend toward better visual acuity improvement with robot-assisted methods. Post-surgery, visual acuity improvements were observed in all 10 eyes. Subfoveal hemorrhage was absorbed in various degrees in all cases. No injection-related complications were observed during the follow-up period. Both injection methods achieved a puncture success rate of 100%. In terms of puncture time (0.31 ± 0.13 min vs 6.50 ± 3.00 min, *P* = 0.003) and subretinal drug injection time (2.66 ± 1.54 min vs 4.48 ± 1.93 min, *P* > 0.05), manual operation efficiency is significantly higher than that of robots (Fig. 7a, b). In terms of POVA, there was no statistically significant difference between the robot-assisted method and the manual method (Fig. 7c). However, the mean of POVA change by the robot-assisted was better than that of the manual method. In addition, the surgical video showed a noticeable tremor in the human hand (see Supplementary Video 6) throughout the later stages of the procedure. The robot-assisted injection system was more stable, and this phenomenon is consistent with the data in Fig. 7d, e. Drift was defined as the total displacement over the entire injection process, and tremor is defined as the amplitude of motion on a small-time scale^35^. The RMS of robot-assisted drift was 41.07 ± 20.78 μm, which was significantly superior to the drift of the manual operation (299.66 ± 85.84 μm). The RMS for robot-assisted tremor is 25.64 ± 15.75 μm, which was better than the tremor of the manual operation (29.98 ± 19.27 μm). In addition, the robot-assisted was able to remain motionless for an extended period of time while injecting a consistent and steady dose of medication. Figure 8 showed an ultra-widefield color image of postoperative visual acuity with the robot. The patient’s visual acuity improved significantly at 4 months after surgery. OCT images (Fig. 9) also demonstrated that foveal hemorrhage subsided in all patients after surgery.

LogMAR (logarithm of the minimum angle of resolution) is a standardized unit used to measure visual acuity, commonly applied in the field of ophthalmology. It provides a way to quantify vision by logarithmically converting the results of visual tests, particularly used to describe visual sensitivity during eye examinations. Smaller LogMAR values indicate better vision, while larger LogMAR values correspond to poorer vision.

PRVA: pre-operative visual acuity, POVA: post-operative visual acuity. Post-operative visual acuity was defined as corrected visual acuity at 2 months after surgery.

## Discussion

Retinal surgery is one of the most delicate and complex surgical tasks. Due to the long period (more than 10 years) of training a qualified retina surgeon and the high demand of the physical condition, excellent retina surgeons have limited for the patients to access, especially in rural areas and limited developing countries. Robot-assisted retinal surgery provides a promising solution to overcome these problems. In this study, a robot-assisted retinal surgical system was proposed and preliminarily verified for clinical treatment for retinal diseases. An RCM parallelogram structure with a double spring adaptive balancing mechanism is designed and optimized to avoid motion interference, reducing the physiological tremor of the surgeon during the operation and achieving precise motion scaling, which significantly improves the precision and stability of the operation. The experimental results show that the motion precision of the SMAR is 0.16°, 0.13°, and 5.56 μm for motion angles *α*, *β*, and *Z*, respectively. The motion ranges of *α*, *β*, and *Z* are 90°, 60°, and 40 mm, respectively. The RCM error of SMAR is 0.15 mm which can achieve safer and less trauma operation compared to manual operation. The deviation from the expected trajectory with manual operation, robot-assisted with balanced device, and robot-assisted without balanced device is 143.06 μm, 26.39 μm, and 61.43 μm, respectively. The results show that the deviation has been significantly reduced by 81.85% from manual to robot-assisted with balanced device operation.

Preliminary human clinical trials showed that the drift of the robot-assisted operation (41.07 μm) was less than the manual operation (299.66 μm). The visual acuity with LogMAR of cases showed higher improvement in the robot-assisted group preliminary, which for manual of 0.78 ± 0.44 vs robot-assisted 1.24 ± 0.70, with no statistically significant difference. It indicates that the robot can perform more delicate operations and reduce the tremor of surgical instruments, which can reduce the trauma and have the potential to improve the outcome of surgery. In addition, the robot can maintain the needle for a long time and inject drugs stably and at a constant speed during the drug injection, which is another advantage of robot-assisted operation. The drawback of robot-assisted surgery is generally slower than manual surgery for all participants. However, the time gap between the robot-assisted and manual groups gets smaller during the number of operations from 14.98 min to 2.87 min, which means that the time is considerably short when the surgeon becomes familiar with the robot system. It shows that the proposed robot can effectively reduce the training curve of surgeons and alleviate the shortage of retina surgeons, which is very important for rural areas and underdeveloped countries. According to the literature^31–33,36,37^, significant progress has been made in the field of ophthalmic surgery robotics. These investigations have advanced from prosthetic testing to clinical applications. The literature^31^ validates the safety and feasibility of intraocular robotic surgery based on a dedicated proof-of-concept device system, with membrane peeling time of 7.33 min. The literature^33^ also designs a miniaturized micro-mechanical hand for microsurgical procedures, inspired by origami, with an accuracy of 26.4 μm, and has been tested on an eyeball phantom for verification. In summary, the proposed method demonstrates superiority.

With the rapid development of medical imaging technology, sensor technology, and robot technology, surgical robots have become an important research direction in the field of robotics. Robot-assisted microsurgery has obvious advantages in operation flexibility, stability, and precision, which promotes the development of surgery in the direction of minimally invasive, intelligent, and precise^38–40^. The further combination of retinal surgical robot technology with micro-force sensing and intraoperative OCT can have the chance to reduce iatrogenic injury, navigate precisely, and improve the outcome of microsurgery^41–43^, making those challenging microsurgeries into more doable and standardize procedures.

## Experimental section

Laboratory Animal Use License Number: SYXK (Su) 2020-0007. The animal welfare involved in this paper complies with the relevant policies and guidelines of this institution. According to the content of this plan, AP is submitted to the Institutional Animal Care and Use Committee (IACUC) for review. AP number: AP-2395-421N. IACUC approval has been obtained before the use of animals in the paper. The clinical trial was approved by the ethics committee of Zhejiang Provincial People’s Hospital (KY2023018).

### Experimental setup and procedure

We characterized the SMAR, which included the SMAR motion precision, RCM error, motion range, and teleoperation test. The camera was fixed in front of the end-effector, which tracked the tip marker of the end-effector and RCM position to analyze the movement of the SMAR. The resolution and frame rate of the video were 1920 × 1080 pixels and 30 frames per second.

### Animal experiment

In all animals, gross observation, optical coherence tomography (OCT), slit lamp (SL), and fundus photography (FP) were carried out on the time point of 1-week pre-operation, 1 day, 2 weeks, and 4 weeks post-operation. Fundus fluorescein angiography (FFA) and electroretinography (ERG) examination were carried out at the time point of 1 week pre-operation, 2 weeks, and 4 weeks post-operation. Intraocular pressure was measured 1 week pre-operation, 3 days, 2 weeks, and 4 weeks post-operation.

Perform bilateral eyeball sampling, dehydration, embedding, sectioning, H.E. staining, mounting, and microscopic examination for all animals. Distinguish between left and right for fixation. Before eyeball extraction, mark the midnight position directly above the eyeball with a suture. Sample three sections of the eyeball, ensuring that the optic disc and the drug administration site are visible in the sections.

### Anesthesia

The first five participants undergoing robot-assisted epiretinal membrane surgery received intra-venous general anesthesia. Upon entering the operating room, atropine was administered intravenously, followed by intravenous injections of propofol, sufentanil, and atracurium cissulfonate for anesthesia induction, muscle relaxation, and mechanical ventilation. Propofol and remifentanil were given intraoperatively to maintain anesthesia depth under the administration of an experienced neuroanesthesia anesthesiologist (A.D.F.). The last five patients receiving subretinal rt-PA injections were provided local anesthesia with a 1:1 mixture of lidocaine and ropivacaine behind the eye without requiring sedation.

### Recruitment and eligibility

The study subjects were recruited from the Department of Ophthalmology, Zhejiang Provincial People’s Hospital. Patients who met the inclusion criteria were assessed by experienced ophthalmologists. All patients signed informed consent forms, and the study adhered to relevant regulations. A total of 10 patients were recruited and randomly assigned to two groups: robot-assisted surgery (*n* = 5) or standard manual surgery (*n* = 5). Additionally, 6 emergency patients with acute submacular hemorrhage were recruited for intravitreal injection of rt-PA, and were randomly divided into the robot-assisted or standard manual surgery groups.

### Training

Before using the robotic system in surgery, all surgical personnel (surgeons, assistants, and nursing staff) underwent a specialized training program. It included the basic knowledge of the device, the operation steps, and the precautions of operation, etc., and carried out virtual reality (VR) simulator training, VR simulator training, and animal live surgery experiments. The clinical operation could not be carried out until all the surgical staff passed the examination.

### Statistical analysis

Origin 2021 native functions were used for data processing. The reported numbers for multiple samples were in the format of “root-mean-square ± standard deviation”. In all box plots, the upper and lower edges of the box represent the 25th and 75th percentiles, the middle line represents the median, and the whiskers extend to the 1.5× interquartile range. For significance analysis, Tukey’s one-way ANOVA test for multiple comparisons was used, *, **, *** corresponding to *P* < 0.05, *P* < 0.01, and *P* < 0.001, respectively.

### Supporting information

Supporting Information is available from the Wiley Online Library or from the author.

## Supplementary information

Overview of the Retinal Surgery

Overview of the SMAR

RCM error assessment

Teleoperation test

Clinical surgery of the SMAR

Clinical assessment of the SMAR

Research highlights

Animal experiment

## Data Availability

All data supporting the findings of this study are available within this paper and its Supplementary Information file. All other relevant data are available from the corresponding author upon request.
