# Supplementary material for: A soft micron accuracy robot design and clinical validation for retinal surgery
Source: Microsyst Nanoeng. 2025 Sep 9;11:170. doi: 10.1038/s41378-025-01002-5 (PMC12420835; doi:10.1038/s41378-025-01002-5)
Supplement: Supplementary file 7 — Research highlights [file 41378_2025_1002_MOESM7_ESM.docx]

- In this research, we present a soft micro accuracy robot (SMAR) for retinal surgery and aim for more stable and safer operation.
- An RCM parallelogram structure with a double spring adaptive balancing mechanism is designed and optimized to avoid motion interference, achieving more precise motion and safer operation. The position precision of the SMAR is 5.56 𝜇𝑚, and trajectory deviation is reduced by 81.85% compared with the human hand.
- Animal and human clinical trials demonstrated that the proposed methods minimize the influence of hand tremors and motion impact on the instrument. Eye surgical robots can help surgeons break through the limits of physiological operation, allowing surgeons to focus more on intraoperative decision-making instead of skills on delicate operation and tremor suppression.
