# Supplementary material for: A soft micron accuracy robot design and clinical validation for retinal surgery
Source: Microsyst Nanoeng. 2025 Sep 9;11:170. doi: 10.1038/s41378-025-01002-5 (PMC12420835; doi:10.1038/s41378-025-01002-5)
Supplement: Supplementary file 8 — Animal experiment [file 41378_2025_1002_MOESM8_ESM.docx]

**Animal experiment**

Ten animals were used for the purpose of comparing the safety and efficacy of subretinal injection in the manual group and robot group. Fast the animals overnight before administration, and the first administration was marked as D1. Compound sodium chloride was a single dose (100μL/eye) subretinal injected manually on the left eye and assisted with the surgical robot on the right eye. Groups and solvent dose settings are shown in Table 1.

**Table 1 Groups and solvent dose.**

| **Group/Color** | **Eye** | **Administration Method** | **Solvent** | **Volume (μL/eye)** | **Animal Number^1，2^** | |
| --- | --- | --- | --- | --- | --- | --- |
|  |  |  |  |  | ♂ | ♀ |
| 1/White  1/White | Left eye | Subretinal injection manually | Compound sodium chloride | 100 | 1001～1005 | 2001～2005 |
|  | Right eye | Subretinal injection assisted with the surgical robot | Compound sodium chloride | 100 |  |  |

**Note:**

Animal numbers consist of 4 digits, where the thousands place indicates gender ('1' for male; '2' for female), and the last two digits indicate the sequence number within the group.

Distribution of duration for subretinal operation in each step including time for operation, time for each puncture, and injection in manual and robot group was recorded and evaluated by reviewing the footage. Time for operation is defined as the period from the moment the injection needle enters the trocar until the needle is withdrawn from the trocar after completing the intraocular operation. The time for each retinal puncture is defined as the period from the moment the injection needle touches the retinal target puncture site until it reaches the target depth. The time for each drug injection is defined as the period from the moment the injection needle punctures into the cavity and injects the drug under the retinal nerve epithelium layer.

In all animals, gross observation, optical coherence tomography (OCT), slit lamp (SL), and fundus photography (FP) were carried out on the time point of one-week pre-operation, one day, two weeks, and four weeks post-operation. Fundus fluorescein angiography (FFA) and electroretinography (ERG) examination were carried out at the time point of one week pre-operation, two weeks, and four weeks post-operation. Intraocular pressure was measured one week pre-operation, three days, two weeks, and four weeks post-operation.

Perform bilateral eyeball sampling, dehydration, embedding, sectioning, H.E. staining, mounting, and microscopic examination for all animals. Distinguish between left and right for fixation. Before eyeball extraction, mark the midnight position directly above the eyeball with a suture. Sample three sections of the eyeball, ensuring that the optic disc and the drug administration site are visible in the sections.
